# Wernicke Encephalopathy Complicating a Distinctive *POLG* Phenotype With MNGIE‐Like Features

**DOI:** 10.1111/ene.70554

**Published:** 2026-03-17

**Authors:** Giuliana Capece, Luca Caumo, Sara Volta, Pietro Riguzzi, Elena Sogus, Angela Petrosino, Sara Vianello, Daniele Sabbatini, Leonardo Salviati, Renzo Manara, Carlo Viscomi, Gianni Sorarù, Luca Bello, Elena Pegoraro

**Affiliations:** ^1^ Neuromuscular Unit, Department of Neurosciences DNS University of Padova Padova Italy; ^2^ Department of Biomedical Sciences University of Padova Padova Italy; ^3^ Department of Cardiac, Thoracic, Vascular Sciences and Public Health University of Padova Italy; ^4^ Department of Women's and Children's Health University of Padova Padova Italy; ^5^ Neuroradiology, Department of Neurosciences DNS University‐Hospital of Padova Padova Italy

**Keywords:** central nervous system diseases, malabsorption syndromes, mitochondrial diseases, mitochondrial encephalomyopathies

## Abstract

**Background:**

Mitochondrial neurogastrointestinal encephalomyopathy (MNGIE) is an extremely rare autosomal recessive disease caused by variants in the thymidine phosphorylase gene (*TYMP*), primarily characterized by severe gastrointestinal and neurological symptoms. The complete phenotype of MNGIE has not been linked to any gene other than *TYMP*.

**Methods:**

We describe two identical twins who exhibited delayed psychomotor development, infantile bilateral cataract, congenital demyelinating polyneuropathy, and severe progressive gastrointestinal dysmotility with recurrent pseudo‐obstruction episodes, along with diffuse supratentorial leukoencephalopathy that mainly overlaps with classic *TYMP*‐related MNGIE. During the course of the disease, one patient developed Wernicke encephalopathy, triggered by chronic malnutrition related to recurrent gastrointestinal pseudo‐obstruction. This patient later suffered from a catastrophic stroke‐like episode, resulting in massive cerebral edema and brain death at the age of 38.

**Results:**

Next‐generation sequencing (NGS) using a custom‐targeted mitochondrial gene panel identified two compound heterozygous variants in the *POLG* gene: the paternal variants p.Thr251Ile and p.Pro587Leu, occurring *in cis*, and the novel maternal variant p.Arg853Gly. Quantification of mtDNA by real‐time PCR on skeletal muscle DNA detected significant depletion, but no multiple deletions were detected with mtDNA analysis by long‐range PCR and Nanopore sequencing.

**Conclusions:**

These cases showed a very distinctive *POLG* phenotype, with some MNGIE‐like features, expanding the clinical and genetic spectrum of the *POLG*‐related diseases. Additionally, they highlighted the importance of monitoring for thiamine deficiency in mitochondrial patients with severe gastrointestinal dysmotility who experience sudden clinical deterioration.

## 1 Introduction

Mitochondrial neurogastrointestinal encephalomyopathy (MNGIE) is a rare, progressive, autosomal recessive, multisystemic disease characterized by ptosis, ophthalmoparesis, peripheral neuropathy, intestinal dysmotility and leukoencephalopathy, typically sparing the corpus callosum [1].

MNGIE is associated with multiple deletions and depletion of mitochondrial DNA (mtDNA) in skeletal muscle and usually caused by imbalance in nucleoside homeostasis due to biallelic variants in *TYMP*, encoding thymidine phosphorylase [1].

Rare patients with MNGIE‐like phenotypes carry variants in genes other than *TYMP* [2, 3, 4]. *LIG3* and *RRM2B* have been linked to incomplete MNGIE phenotypes. With *LIG3* variants, leukoencephalopathy was mild and involved the corpus callosum, with epilepsy and stroke‐like episodes (unusual with *TYMP* variants) [3]. Similarly, with *RRM2B* variants, the periventricular‐subcortical leukoencephalopathy was patchy, with symmetric basal ganglia hyperintensities (atypical for classical MNGIE) [4].

Gastrointestinal involvement is common in *POLG*‐related diseases, where an incomplete MNGIE‐like phenotype, lacking leukoencephalopathy and demyelinating neuropathy, has been reported in 3.3% of patients with *POLG* variants [2, 5].

Human mtDNA is replicated by polymerase gamma, composed of a catalytic subunit, encoded by *POLG*, and two accessory subunits. The mature protein consists of an exonuclease domain with proofreading activity, a linker domain, and a polymerase domain [6].

We report on two identical twins with a MNGIE‐like phenotype, harboring compound heterozygous *POLG* variants.

## 2 Methods

### 2.1 Patients

Patients #1 and #2 are identical male twins born to healthy, unrelated Italian parents, with no family history of neurological disorders. Both experienced delayed psychomotor development and mild intellectual disability. At the age of 3, they were diagnosed with sensorimotor demyelinating polyneuropathy. A muscle biopsy performed on patient #1 at that time showed increased subsarcolemmal red‐staining material in a few fibers (modified trichrome stain) and neurogenic atrophy with type 1 fiber predominance.

### 2.2 Clinical Course and Follow‐Up

Both patients underwent bilateral cataract removal and developed progressive muscle weakness, especially in the distal lower limbs. At age 12, patient #1 underwent bilateral Achilles tenotomy. In early childhood, both developed dyspeptic symptoms (abdominal pain, nausea, early satiety, and post‐prandial fullness), which evolved into severe gastrointestinal dysmotility and recurrent episodes of pseudo‐obstruction, ultimately leading to malabsorption, weight loss, and repeated hospitalizations. Patient #2 required percutaneous endoscopic gastrojejunostomy (PEG‐J) placement at age 35.

### 2.3 Neurological Evaluation (Age 35)

**FIGURE 1:**
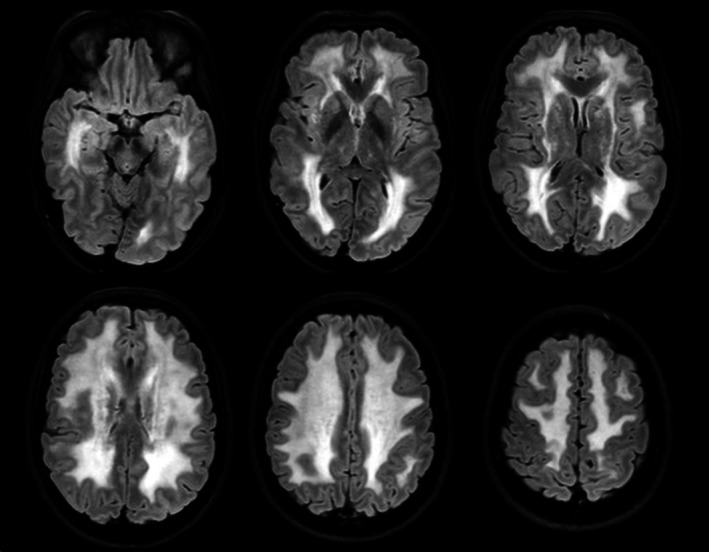
Neuroradiological findings. Axial T2 fluid‐attenuated inversion recovery imaging (FLAIR) shows diffuse, confluent and symmetric severe supratentorial white matter hyperintensities with sparing of U fibers and corpus callosum.

At presentation, both patients had a BMI of 17. Neurological examination revealed cerebellar and sensory ataxia, bilateral foot drop, diffuse muscle atrophy and weakness (more severe distally), and absent deep tendon reflexes. Both had decreased fine touch and vibratory sensation, bilateral ptosis, ophthalmoparesis, and nasal speech. Brain MRI showed severe, symmetrical supratentorial T2 white matter hyperintensity with sparing of U‐fibers and the corpus callosum (Figure 1). Cardiac function was normal, while inspiratory and expiratory muscle strength was reduced with normal arterial blood gases. Bilateral mild sensorineural hearing loss was noted.

### 2.4 Genetic and Biochemical Testing

*TYMP* gene sequencing was normal. NGS with a 520‐gene mitochondrial panel revealed a complex allele with compound heterozygous variants in *POLG*: c.[752C>T + 1760C>T]/p.[Thr251Ile + Pro587Leu] (mapping to the exonuclease and linker domains), and a novel variant c.2557C>G, p.Arg853Gly in the DNA‐binding region of the catalytic domain, with segregation confirming its maternal origin.

### 2.5 Muscle Tissue Analysis (Patient #1)

Complex I activity: 5.1 nmol/min/mg (normal: 15.0–25.6); complex IV activity: 1.5 nmol/min/mg (normal: 30.3–51.1) [7]; mtDNA quantification (real‐time PCR): ~10% of age‐ and sex‐matched controls (Table S1). Long‐range PCR and Nanopore sequencing: no evidence of multiple deletions.

## 3 Results

**FIGURE 2:**
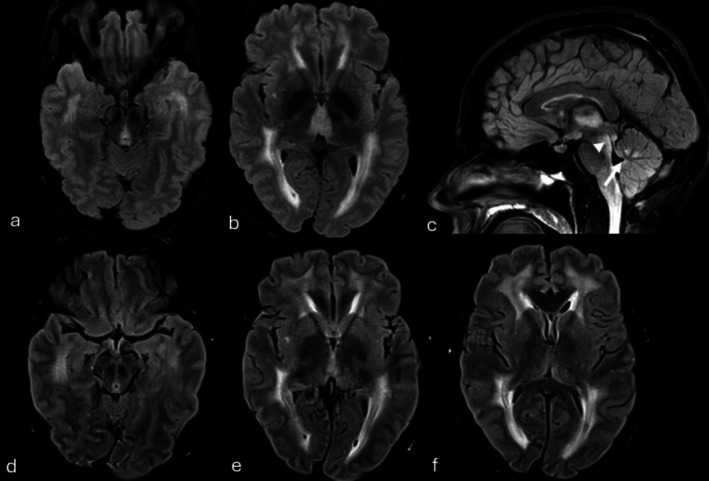
Wernicke's encephalopathy. Axial (a, b) and midsagittal (c) FLAIR images show bilateral symmetrical hyperintensity in the medial thalamus, mammillary bodies, periaqueductal gray matter and inferior colliculi (c, arrowhead), cerebellar vermis (c, white arrow), consistent with Wernicke encephalopathy. A one‐month follow up MRI shows the pre‐existent severe supratentorial leukoencephalopathy while the Wernicke's encephalopathy related changes had disappeared (d–f).

At age 36, patient #1 was admitted to the Neurology Unit due to acute worsening of muscle weakness, hearing loss, decreased visual acuity, headache, and new‐onset diplopia following a gastrointestinal pseudo‐obstruction episode with vomiting and hyporexia. Blood lactate was elevated at 9.8 nmol/L (normal: 0.5–2.2). Brain MRI revealed new bilateral T2‐hyperintense lesions in the medial thalami, mammillary bodies, periaqueductal gray matter, cerebellar vermis, and quadrigeminal plate, in addition to the pre‐existing leukoencephalopathy (Figure 2). Wernicke's encephalopathy (WE) was suspected, and intravenous thiamine supplementation was promptly initiated. Pre‐treatment thiamine level was 31 nmol/L (normal: 66–200). Neurological symptoms resolved rapidly. One‐month follow‐up MRI showed resolution of the new lesions with persistence of the prior white matter abnormalities.

Approximately two years later (age 39), patient #1 was admitted to the Emergency Department after a transient loss of consciousness followed by global aphasia and confusion. The day before, he had reported a headache. Emergency personnel promptly arrived and found no evidence of major cardiac abnormalities. The patient was responsive and breathing normally, with no need for resuscitation. On admission, about 80 min later, his vital signs were normal, and no rhythm abnormalities or indirect signs of impending heart failure were detected, neither by the helicopter paramedics nor in the emergency room or later in the intensive care unit. Neurological examination revealed mild global aphasia, left‐sided mild mydriasis, and severe psychomotor agitation. Brain CT (performed within 10 min of admission) showed no acute findings other than the pre‐existing leukoencephalopathy. He subsequently developed myoclonic jerks and rapidly deteriorated into coma (GCS 7: E4V1M2), requiring orotracheal intubation, while continuous hemodynamic monitoring consistently demonstrated stable vital signs. Brain MRI (three hours after admission) demonstrated severe cytotoxic brain edema (ADC values: ~200 × 10^−6^ mm^2^/s in white matter; ~450 × 10^−6^ mm^2^/s in gray matter), signs of critical intracranial hypertension, and absent intracranial arterial flow on MR‐angiography (Figure S1). Brain death was declared the following day. Blood lactate and thiamine levels were normal.

At the last follow‐up (age 44), patient #2 showed significant worsening of limb and truncal ataxia, along with more frequent pseudo‐obstruction episodes.

## 4 Discussion

The clinical presentation of our patients is unique within the spectrum of *POLG*‐related disorders [2, 8]. To the best of our knowledge, a clinical syndrome resembling the typical *TYMP*‐related MNGIE phenotype, with brain leukoencephalopathy (sparing the U‐fibers and corpus callosum) and demyelinating neuropathy, has been documented in rare cases. Two patients, carrying compound heterozygous *POLG* variants, had very mild, non‐specific white matter abnormalities [9, 10], while two Tunisian boys, carrying a pathogenic *OPA1* variant, also had optic atrophy [11]. However, *TYMP*‐related and *POLG*‐related MNGIE usually show different progression.

In our cases, severe gastrointestinal symptoms appeared in adulthood, preceded by dyspeptic symptoms in early childhood, whereas congenital demyelinating neuropathy and early cataracts were diagnosed in infancy.

Interestingly, a case of a MNGIE‐like phenotype with early childhood bilateral cataracts was reported, but with normal brain MRI findings and only one *POLG* variant [12].

A unique complication of gastrointestinal involvement in *POLG* disease occurred in patient #1, who developed WE, due to thiamine deficiency. Mitochondrial disorders may predispose patients to WE, since thiamine is a cofactor in carbohydrate metabolism and energy production and WE‐like syndrome has been previously reported in a *TYMP*‐related MNGIE [13, 14].

In our case, pre‐existing *POLG* disease and chronic gastrointestinal dysfunction likely contributed to thiamine depletion, which was exacerbated by recurrent vomiting and malnutrition. This case underscores the need for high clinical suspicion for WE in mitochondrial patients with gastrointestinal involvement. Early recognition and prompt intravenous thiamine replacement are essential, and long‐term oral supplementation with regular thiamine monitoring should be considered.

The cause of death in patient #1 remains uncertain. He presented with headache, followed by loss of consciousness, transient aphasia, and confusion—symptoms suggestive of a focal seizure, a well‐established trigger of stroke‐like episodes. The subsequent rapid neurological deterioration in an otherwise hemodynamically stable patient may reflect either a fulminant evolution of a stroke‐like episode or metabolic decompensation secondary to mitochondrial dysfunction and energy failure. Cardiac arrhythmias appear unlikely, given continuous electrocardiogram monitoring, and hepatic involvement is improbable due to normal liver function. Although stroke‐like episodes are hallmarks of MELAS syndrome, they have also been reported in association with *POLG* variants [6].

By contrast, patient #2 is still alive at age 44, showing relatively long survival despite early disease onset, although late‐onset *TYPM*‐related MNGIE have been described [15].

Both patients were compound heterozygous for p.[Thr251Ile + *p*.Pro587Leu] and p.Arg853Gly *POLG* variants. The latter is a novel variant, reported only once over one million alleles in the GnomAD v4.1 database, and classified as “Likely pathogenetic” in ClinVar (ID: 2778434). Arginine 853 is a highly conserved residue within the polymerase domain. Other missense substitutions at this position (e.g., p.Arg853Trp and p.Arg853Gln) have been reported in association with severe mitochondrial disorders, including myocerebrohepatopathy spectrum disorders [6], progressive external ophthalmoplegia [16], and juvenile onset parkinsonism with axonal neuropathy [17]. According to ACMG criteria, p.Arg853Gly can be classified as “Likely Pathogenic.”

The complex allele p.[Thr251Ile + *p*.Pro587Leu] is one of the most common recessive variants in *POLG*, accounting for 6% of variants in unrelated affected patients and found heterozygously in ~1% of the Italian population [18]. The p.Thr251Ile and the p.Pro587Leu, located in the exonuclease and linker domains respectively, are always found *in cis* and are thought to act synergistically by impairing DNA binding, reducing polymerase thermostability, and diminishing exonuclease activity. Together, these effects severely reduce polymerase function (~5% of normal activity) [19].

In conclusion, *POLG*‐related disorders should be strongly considered in the differential diagnosis of atypical MNGIE without *TYMP* variants. To the best of our knowledge, this is the first report of a very distinctive *POLG*‐related phenotype mainly overlapping MNGIE syndrome. The novel *POLG* variant p.Arg853Gly further broadens the genetic and clinical spectrum of *POLG*‐associated disorders.

This report also highlights WE as a potentially reversible yet life‐threatening complication in mitochondrial patients with gastrointestinal involvement.

## Author Contributions

Clinical data collection: Elena Pegoraro, Giuliana Capece, Luca Caumo, Pietro Riguzzi, Angela Petrosino, and Elena Sogus. Genetic and biochemical analysis: Sara Vianello, Sara Volta, Leonardo Salviati, and Carlo Viscomi. Neuroimaging: Renzo Manara. Writing‐original draft: Giuliana Capece and Luca Caumo. Writing‐review and editing: all co‐authors. Elena Pegoraro is the study guarantor. All authors read, critically revised, and approved the final manuscript.

## Funding

This work was supported by Fondazione IRP Città della Speranza, Fondazione Telethon (Grants GGP20013 and GTB12001D), Associazione Luigi Comini Onlus.

## Disclosure

Carlo Viscomi holds a patent [PCT/IB2022/052275], he received support for attending meetings and/or travel from Associazione Luigi Comini Onlus and he participated in advisory boards for EU‐Marie Curie in the Scientific advisory board of the NADIS project. He participated in Mitocon Scientific board. Mitocon provided Oroboros Next Generation O2K equipment. The other authors have nothing to report.

## Ethics Statement

The Medical Ethical Committees “Comitato Etico Territoriale Area Centro‐Est Veneto” reviewed the manuscript and raised no objections to its submission.

## Conflicts of Interest

The authors declare no conflicts of interest.

## Supporting information


**Table S1:** Biochemical and molecular analysis of the patient's skeletal muscle. (A) Spectrophotometric analysis of the respiratory chain. (B) Analysis of mtDNA content by real‐time PCR.
**Figure S1:** Massive stroke‐like episode. Axial diffusion weighted imaging (DWI) sequences (a, b and c) and FLAIR sequences (d, e and f) show diffuse cytotoxic and vasogenic edema with obliteration of sulcal spaces and compression of the ventricles.

## Data Availability

Data is provided within the manuscript or Supporting Information files. Further data are available from the corresponding author, upon reasonable request.
