# Supplementary material for: Wernicke Encephalopathy Complicating a Distinctive POLG Phenotype With MNGIE‐Like Features
Source: Eur J Neurol. 2026 Mar 17;33(3):e70554. doi: 10.1111/ene.70554 (PMC13093735; doi:10.1111/ene.70554)
Supplement: Supplementary file 1 — Table S1: Biochemical and molecular analysis of the patient's skeletal muscle. (A) Spectrophotometric analysis of the respiratory chain. (B) Analysis of mtDNA content by real‐time PCR. Figure S1: Massive stroke‐like episode. Axial diffusion weighted imaging (DWI) sequences (a, b and c) and FLAIR sequences (d, e and f) show diffuse cytotoxic and vasogenic edema with obliteration of sulcal spaces and compression of the ventricles. [file ENE-33-e70554-s001.docx]

**Supporting Information**

**Wernicke encephalopathy complicating a distinctive *POLG* phenotype with MNGIE-like features.**

Giuliana Capece, MD^1*^, Luca Caumo, MD^1*^, Sara Volta^2^, Pietro Riguzzi, MD^1^, Elena Sogus MD^1^, Angela Petrosino MD^1^, Sara Vianello, PhD^1^, Daniele Sabbatini, PhD^3^, Leonardo Salviati, MD^4^, Renzo Manara, MD^5^, Carlo Viscomi, PhD^2^, Gianni Sorarù MD PhD^1^, Luca Bello MD, PhD^1^, Elena Pegoraro MD PhD^1^

^1^ Neuromuscular Unit, Department of Neurosciences DNS, University of Padova, Padova, Italy

^2^Department of Biomedical Sciences, University of Padova, Padova, Italy

^3^Department of Cardiac, Thoracic, Vascular Sciences and Public Health, University of Padova, Italy

^4^Department of Women's and Children's Health, University of Padova, Padova, Italy

^5^Neuroradiology, Department of Neurosciences DNS, University-Hospital of Padova, Padova, Italy

*these two authors equally contributed to the study and should be considered first coauthors

Corresponding Author:

Elena Pegoraro, MD PhD

Department of Neurosciences DNS, University of Padova, via Giustiniani, 5, 35128 Padova, Italy

Phone: + 39 049 8213622

Fax: + 39 049 8751770

ORCID: 0000-0002-7740-4156

e-mail: elena.pegoraro@unipd.it

**Biochemical and molecular findings**

Mutations in *POLG* are associated with mtDNA instability and defects in oxidative phosphorylation (OXPHOS). We thus measured the spectrophotometric activities in patient #1 and found reduced complex I and IV (Supplementary Table S1A). Quantification of mtDNA by real time PCR on skeletal muscle DNA detected profound depletion compared to an age and sex matched control (Supplementary Table S1B). MtDNA analysis by long-range PCR and Nanopore sequencing did not detect the presence of multiple deletions.


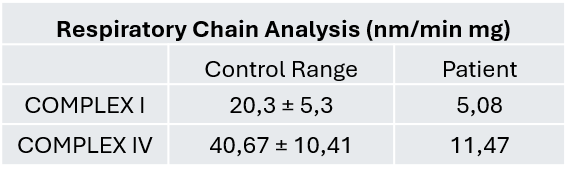


**A.**


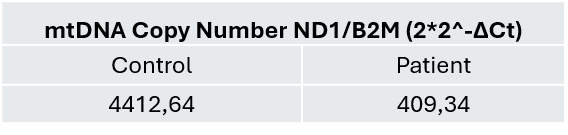


**B.**


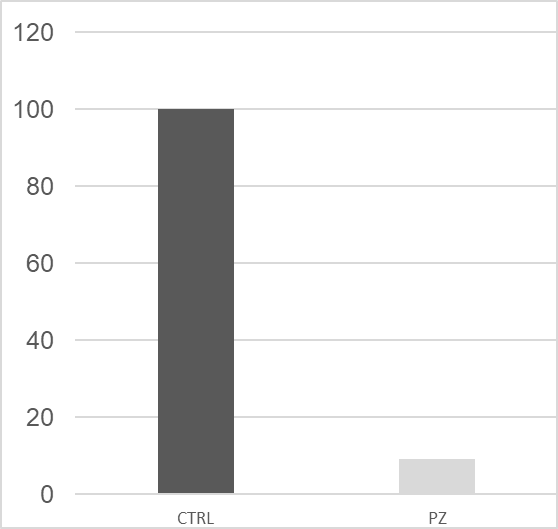


mtDNA

**Table S1. Biochemical and molecular analysis of the patient’s skeletal muscle.** (A) Spectrophotometric analysis of the respiratory chain. (B) Analysis of mtDNA content by real-time PCR.


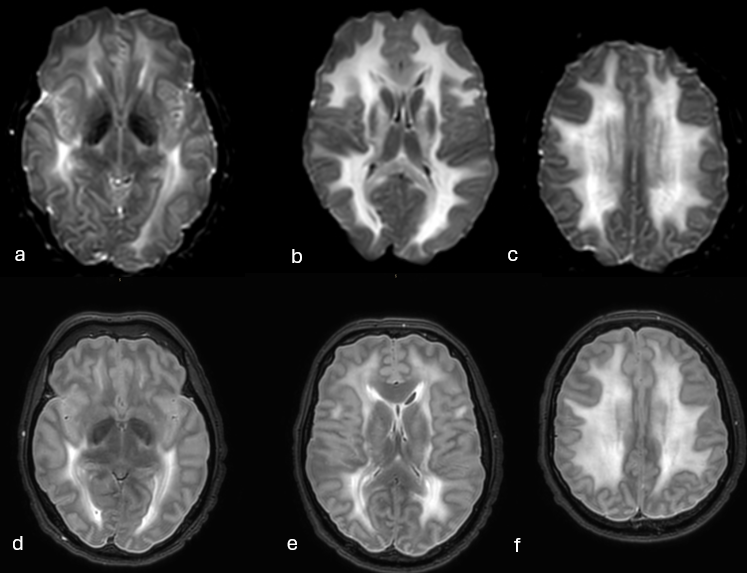


**Figure S1. Massive stroke-like episode.** Axial diffusion weighted imaging (DWI) sequences (a, b and c) and FLAIR sequences (d, e and f) show diffuse cytotoxic and vasogenic edema with obliteration of sulcal spaces and compression of the ventricles.
